# Materials for sustainable energy production, storage, and conversion

**DOI:** 10.3762/bjnano.6.163

**Published:** 2015-07-23

**Authors:** Maximilian Fichtner

**Affiliations:** 1 Helmholtz Institut Ulm (HIU), Helmholtzstraße 11, 89081 Ulm, Germany

A steadily growing human population and the growing global economy have led to increasing energy consumption and the realization that fossil fuels are a finite resource on earth. Moreover, due to the rising global temperature, there is a need to reduce CO_2_ emissions to mitigate or prevent further global warming. Both needs have fueled international efforts to convert the current energy supply strategy into an economy which must eventually be based on renewable energy sources such as solar, wind, biomass, geothermal, and water power. Hence, these factors have driven the intensified and ever-growing interest in the fields of energy harvesting and storage seen during the past two decades.

The harvesting of light is still a challenge, and solutions are needed to achieve both high efficiency and low cost electricity generation. While wind and sun are among the most powerful options for electricity generation in general, their intermittent nature makes large storage capacity necessary. Storage is also needed to balance electricity grids and to avoid overcapacity.

A particular challenge is the storage of electrical energy for stationary, mobile, and portable applications. Here, solutions are needed that are based either on chemical compounds, such as hydrogen or hydrocarbons, or on safe, low cost and powerful batteries, which have a long cycle and calendar life and a high energy density. At the same time, any long term option for energy storage must be based on sustainable materials involving abundant elements in the Earth’s crust. For the reconversion of hydrogen or organic liquids (energy carriers), efficient fuel cells are needed as converters, preferably those based on non-noble metals with a long lifetime and a low kinetic barrier for conversion. For electrochemical storage, batteries based on new ionic shuttles such as sodium or magnesium are being explored. Moreover, oxygen from air could serve as an active cathode material, which does not need to be intrinsically integrated in the cell.

In addition to the harvesting and storage of electrical energy, the storage of heat is another essential element in the future energy landscape. Thus, the heating of homes and the efficiency of industrial processes will greatly benefit from the availability of cost efficient, heat storage devices.

This Thematic Series collects selected contributions from the last Materials for Energy Conference (ENMAT 2013) in Karlsruhe, Germany, which addressed important research topics in the abovementioned fields. The articles summarize the state-of-the art in the field, give perspectives, and present recent results from the respective working groups.

In the field of energy harvesting and photovoltaics, Benedikt Iffland and Christian Jooss [1] report on current–voltage characteristics of manganite–titanite perovskite junctions. State-of-the art and recent progress in energy conversion from chemical carriers are discussed in two contributions covering materials issues in two different types of fuel cells: Gregorii L. Soloveichik reports on challenges and perspectives in the field of liquid fuel cells [2]. Materials issues in polymer electrolyte membrane fuel cells operating at moderately elevated temperatures (HT-PEMFC) below 200 °C are discussed by Roswitha Zeis [3].

The field of electrochemical energy storage is particularly challenging. Current Li-ion batteries are not only expensive and have a relatively short lifetime, they are also considered to not have enough energy content to meet the demands of future applications. Efficient systems based on powerful and sustainable materials beyond lithium are needed in order to provide long term solutions. In this respect, three contributions were selected from Rana Mohtadi, Luc Aymard, and Philip Adelhelm [4–6], presenting the progress on novel systems involving Mg batteries, conversion electrodes based on hydrides, and Na and Li air batteries, respectively. In the fields of fuel cells and batteries, multiscale theoretical modeling is considered to be essential to both understand the structures and energetics of energy materials as well as their function when integrated into a battery electrode. Not only can a better understanding of the transport processes and chemical reactions at the microscale be gathered, but also the development of strategies for optimizing the electrode. Such a multiscale modeling approach is presented with examples in the contribution by Arnulf Latz and Jochen Zausch [7]. Last but not least, Nicole Pfleger, Antje Wörner and colleagues [8] discuss the current state-of-the art and future options for thermal storage using nitrate salts.

I would like to thank all authors and the referees for their effort and excellent contributions. Special thanks go to the team at the Beilstein Journal of Nanotechnology for their continuous support in the handling of this series. The open access policy of the Beilstein-Institut was a strong motivation for both the editor and the authors of this series to contribute, as colleagues from all over the world are able to freely access the contributions in this journal. Finally, I would like to thank the DECHEMA for their strong and highly professional support in realizing the ENMAT conference.

Maximilian Fichtner

Ulm, June 2015
